# Uplift modeling to determine which fluid-norepinephrine regime results in a postoperative acute kidney injury-free recovery in patients scheduled for cystectomy and urinary diversions

**DOI:** 10.3389/fmed.2025.1542797

**Published:** 2025-06-11

**Authors:** Markus Huber, Marc A. Furrer, François Jardot, Patrick Y. Wuethrich

**Affiliations:** ^1^Department of Anaesthesiology and Pain Medicine, Inselspital, Bern University Hospital, University of Bern, Bern, Switzerland; ^2^Department of Urology, Inselspital, Bern University Hospital, University of Bern, Bern, Switzerland; ^3^Department of Urology, Solothurner Spitäler AG, Olten, Switzerland

**Keywords:** acute kidney injury, hemodynamic management, cystectomy, urinary diversion, machine learning, prediction modeling, heterogeneous treatment effects

## Abstract

**Background:**

Postoperative acute kidney injury (PO-AKI) remains common after surgery. Although risk prediction models for PO-AKI exist, it is still unknown which intraoperative regime in terms of fluid and norepinephrine administration is beneficial for a specific patient. We thus aim to investigate the potential of uplift modeling—a framework combining causal inference and machine learning—in identifying patients for which certain fluid and norepinephrine regimes result in a PO-AKI-free recovery.

**Methods:**

Data from a prospectively maintained cystectomy database at a single tertiary center (*N* = 1,482, period 2000–2020) were used. Total intraoperative fluid balance (TIFB) and norepinephrine (NE) administration were dichotomized into a high TIFB/low NE and a low TIFB/high NE regime. Primary outcome was PO-AKI. Confounding was addressed with inverse probability of treatment weighting. Uplift was defined as the difference in likelihood of no PO-AKI with a high TIFB/low NE versus low TIFB/high NE treatment regime. We modeled uplift using logistic regression and random forests as outcome models. Model performance was evaluated with the area under the Qini curve (AUQC).

**Results:**

The uplift models demonstrated a higher ability (AUQC: 0.30, 95%-CI: 0.26–0.30) compared to a random sorting strategy (0.06, 95%-CI: 0.02–0.06) or a traditional prediction model (AUQC: 0.06, 95%-CI: 0.03–0.06) for PO-AKI in sorting patients according to the expected treatment benefit from either a high TIFB / low NE or a low TIFB / high NE regime. The performance of the uplift models is robust with respect to the fluid-NE dichotomization.

**Conclusion:**

Uplift modeling provides a clinically relevant step toward personalized medicine by considering the incremental benefit of an alternative treatment versus a control treatment on a patient’s outcome, thus moving from a predictive toward a prescriptive risk assessment. We demonstrated the overall higher clinical utility of an uplift modeling approach compared to a prediction model of baseline PO-AKI risk in sorting patients according to the expected treatment benefit from either a high total intraoperative fluid balance / low norepinephrine regime or a low total intraoperative fluid balance / high norepinephrine regime with respect to postoperative acute kidney injury.

## 1 Introduction

Despite advances in perioperative safety over the past decades, postoperative acute kidney injury (PO-AKI) remains a common complication after major surgery (1, 2). A recent multinational study highlighted that about one in five patients develops PO-AKI (3). Importantly, PO-AKI is associated with worse patient outcomes, e.g., with longer hospital stay and higher mortality (4, 5). Thus, identifying patients at high risk of PO-AKI and diagnosing it early remain major goals for a reduction of the perioperative burden of AKI (6). Clinical prediction models, risk scores and nomograms for the likelihood of PO-AKI exist and provide valuable information for risk stratification (7–9). Despite their clinical utility, these risk prediction frameworks do not allow to provide decision support for the clinician in terms of suitable treatment options for individual patients. Such causal decision-support would require a consideration of the causal relationships underlying treatment options, risk factors and the outcome of interest (10).

To achieve this goal, however, one needs to move from a purely predictive risk approach toward a prescriptive approach that puts the causal effect of a treatment on the outcome of interest and the heterogeneity of patients’ responses to treatments center stage (11–13). The term prescriptive refers to the notion that one actively considers the causal effects of different treatment options on a patient’s outcome. In this context, the investigation and modeling of heterogeneity of treatment effects (HTEs) using data from randomized controlled trials (RCTs) has gained traction over the past years to overcome the limitations of summary RCTs results and subgroup analyses and to provide clinically relevant decision support for patient-centered care (14, 15). The aim of predictive HTE is to develop models that can be used to predict which of two or more treatments will be best for individual patients when taking into account multiple variables influencing the treatments’ benefits or harms (16).

Initially coined in marketing research—where the term uplift refers to the response rate difference between two randomized groups—uplift modeling follows the spirit of predictive HTE and focuses on the incremental effect of a treatment with respect to a control treatment on the unit of interest (17). In the clinical domain and in contrast to prognostic targeting of patients—where the focus traditionally lies on patients with high baseline risks—uplift modeling attempts to identify those patients who benefit from a particular drug or intervention (18, 19).

Therefore, we aim to build and evaluate multiple uplift models to identify patients who would benefit from a particular perioperative fluid-norepinephrine regime. A short introduction into uplift modeling is provided and differences in model evaluation between predictive and prescriptive modeling are illustrated. We describe how the traditional uplift approach based on data from a randomized controlled trial was adapted to an observational dataset. We conclude by highlighting the clinical importance and applicability of such an uplift modeling approach in clinical practice.

## 2 Uplift modeling

### 2.1 Definition

In essence, uplift modeling features methods and notions both from Causal Inference and Machine Learning as it comprises the potential outcome framework as well as outcome prediction methods (20, 21). The term uplift is defined as the likelihood of a beneficial outcome with an alternative (or new) treatment with respect to the control (or standard) treatment. The objective of uplift modeling is to derive a sorting mechanism—or patient identification model—that is able to sort patients according to the expected benefit from the alternative treatment with respect to the control (or standard) treatment. Such sorting mechanisms—and the associated subgroups resulting from the sorting—are highly relevant for clinical practice, e.g., in cases where the costs of a new, superior drug or intervention are high or resources are limited (12, 22).

We start by first considering the uplift modeling framework for the case of a RCT. Subsequently, we illustrate how we extended the RCT-based uplift framework to accommodate observational study data.

### 2.2 Randomized controlled trial data

**FIGURE 1:**
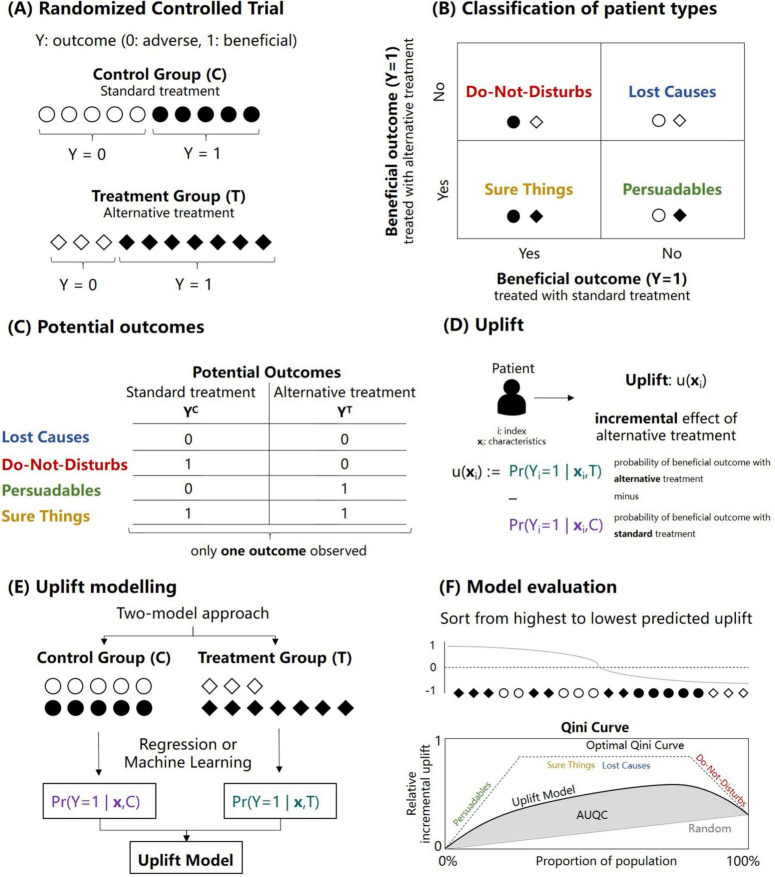
Illustration of the concept of uplift modeling for sorting patients according to expected treatment benefit. **(A)** Outcomes of a two-arm randomized controlled trial with a control (C) and treatment group (T) and a binary outcome Y. Note that the outcome with label “1” refers to the beneficial outcome. **(B)** Classification of possible response types into four categories: Sure things, Lost causes, Do-not-disturbs and Persuadables. **(C)** Non-observable potential outcomes associated with the four response types. **(D)** Definition of the term upflift which refers to the incremental treatment effect of the alternative treatment (denoted here as treatment T) with respect to the standard treatment (denoted here as control treatment C) for a patient *i* with covariates *x*_*i*_. **(E)** Illustration of the so-called two-model approach to calculate the uplift for each patient. The approach is based on fitting two prediction models (e.g., based on regression or machine learning methods) separately for the control and treatment group. **(F)** Model evaluation of an uplift model based on sorting the predicted uplift values in a descending fashion. After the sorting, the relative incremental uplift is calculated for different proportions of the population, resulting in the so-called Qini curve from which the area under the Qini curve can be calculated (AUQC; see Materials and methods).

The case of a RCT is the standard case for the computation of individualized treatment effects, which are at the center of uplift modeling (21). Figure 1 illustrates the concept of uplift modeling when data from a RCT is used—note that in contrast to the standard convention often seen in the risk prediction literature, the outcome is coded in such a way that a beneficial outcome is coded as 1 and the adverse outcome as 0 (Figure 1A). The patients participating in the trial can be classified according to four response types: *Sure Things*, *Lost Causes*, *Do-not-disturbs* and *Persuadables* (Figure 1B). In epidemiology, these four types are also referred to as *Doomed*, *Saved*, *Harmed* and *Immune* (i.e., when an adverse outcome is considered) (11). For example, a patient labeled as *Sure Thing* would experience a beneficial outcome irrespective of the treatment. The clinically relevant concept of response types is based on different values of the potential outcomes (Figure 1C). The response types are not observable as only one of the two potential outcomes can be observed for each patient, reflecting the so-called fundamental problem of causal inference (23).

Figure 1D illustrates the definition of the term upflift: uplift refers to the (unobserved) incremental treatment effect of the alternative treatment (denoted here as treatment T) with respect to the standard treatment (denoted here as control treatment C) for a patient *i* with covariates *x*_*i*_ (e.g., baseline characteristics and comorbidities). A positive uplift value suggests a beneficial treatment effect (with respect to the control treatment). In contrast, a negative uplift value suggests that the patient benefits from the control treatment. Uplift thus reflects the estimate of Conditional Average Treatment Effect (CATE). The so-called two-model approach to calculate the uplift for each patient is shown in Figure 1E: the approach is based on fitting two outcome prediction models (e.g., based on regression or machine learning methods) separately for the control and treatment group (22, 24). There are other statistical approaches to uplift model building; however, we focus here only on the two-model approach for simplicity and we refer the reader to the literature for more advanced uplift frameworks (13, 19).

Note that the four response types (*Sure Things*, *Lost Causes*, *Do-not-disturbs*, and *Persuadables*) can be derived by dichotomizing the probabilities of the two prediction models on which the two-way uplift model is based (Figure 1). For example, setting the likelihood of a beneficial outcome with the alternative treatment to 1 if the likelihood is above 0.5 and similarly for the control treatment, the response type for a patient can be derived. A detailed example is illustrated in the Supplementary material. The focus in the remaining part of the study, however, lies in continuous uplift values as the full probabilistic information from the uplift model is lost when only the four response types are investigated.

### 2.3 Uplift model evaluation

The evaluation of an uplift model deserves special consideration. In traditional response modeling, the probabilistic predictions of a model can be compared to the actual observations of an independent cohort, for example, within an internal cross-validation framework or using an external validation cohort. Since the uplift is not observable, traditional model evaluation metrics are not feasible as there is no ground truth to which the uplift predictions can be compared to (25). The solution is to resort to aggregated (or group-level) metrics for uplift model evaluation as illustrated in Figure 1F. First, patients are sorted in a descending fashion according to the predicted uplift. After the sorting, the relative incremental uplift is calculated for different (cumulative) proportions of the population, resulting in the so-called Qini curve. For a specific quantile *p* with respect to the sorted uplifts, the Qini curve as a function of the (sorted) quantiles—denoted as q(*p*)—is defined as (20, 26):


$$q\,\left(p\right)=Y_{p}^{T}-\frac{Y_{p}^{C}\,N_{p}^{T}}{N_{p}^{C}},$$

where $Y_{p}^{T}$ refers to the sum of the beneficial outcomes in patients in the treatment group (whose predicted uplift is part of the specific quantile *p*), $Y_{p}^{C}$ refers to the corresponding sum of the beneficial outcomes in patients in the control group and $N_{p}^{T}$ and $N_{p}^{C}$ refer to the number of patients in the treatment and control group with respect to the specific quantile. The resulting Qini curve and its associated area under the Qini curve (AUQC) are illustrated in Figure 1F. Similar to the area under the receiver operating characteristic curve (AUROC), the AUQC provides a useful evaluation metric to compare different uplift models.

As the overall objective of uplift modeling is patient identification by sorting the patients according to the expected treatment benefit, there are two benchmark scenarios to which the AUQC of a particular uplift model can be compared to. First, the AUQC can be calculated for the case of random patient sorting. An uplift model with actual clinical utility should feature an AUQC larger than the AUQC derived from a random sorting strategy. The second benchmark scenario refers to an idealized sorting strategy, in which it is assumed that all those patients with a beneficial outcome constitute the *Persuadable* response type and that these patients are identified first (Figure 1F). Subsequently, this idealized sorting mechanism would then identify subsequently the *Sure Things* and *Lost Causes*, and finally the *Do-not-disturbs*. Although idealized, this benchmark scenario gives an indication of how large the AUQC for an actual uplift case could be—similar to the upper boundary of 1 for the AUROC.

The so-called uplift by decile plot constitutes an additional model evaluation tool (20). First, the uplift is predicted for each patient. Second and for the control and treatment group separately, patients are sorted with respect to the predicted uplift and grouped according to deciles. Third, the average uplift is computed in each decile and the difference between the control and treatment group is calculated for each decile. Plotting the differences with respect to the decile allows to examine if the model predicts a high uplift in the top decile and a low uplift in the bottom deciles. In the ideal case, the uplift by decile plot shows a monotonically decreasing curve from the top decile (0–10%) to the bottom decile (90–100%) with respect to predicted uplift (27).

### 2.4 Observational data

**FIGURE 2:**
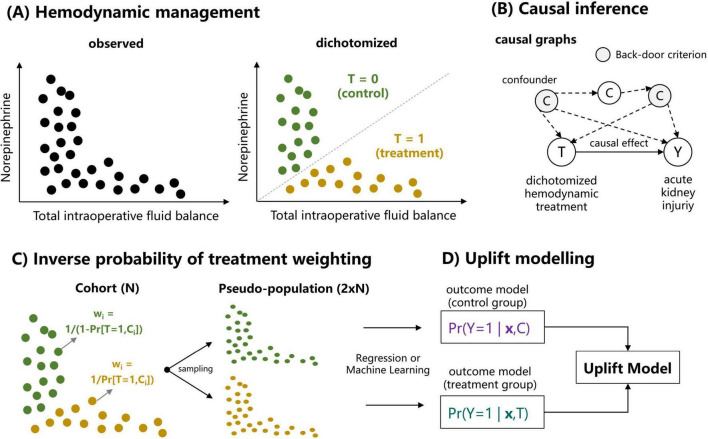
Illustration of deriving an uplift model relating hemodynamic management and the primary outcome (postoperative acute kidney injury) for an observational dataset. **(A)** Dichotomization of the two continuous treatment variables [total intraopoerative fluid balance (TIFB) and norepinephrine administration (NE)] into a binary treatment variable referring to the case of a high TIFB and low NE (referred to as treatment, T = 1) and low TIFB with high NE (referred to as control, T = 0). **(B)** The causal effect of the dichotomized hemodynamic treatment (T) on postoperative acute kidney injury (Y) is based on a causal graph and is identified by means of back-door criterion (30). **(C)** Using inverse probability of treatment weighting (IPTW) and associated weights, a pseudo population is sampled in which the set of confounding variables identified by the backdoor-criterion are balanced. **(D)** Based on the sampled pseudo population, outcome models for the control and treatment group are computed to build an uplift model (see Figure 1 and Supplementary Figure SM1).

In observational data, the control and treatment group are (potentially) non-exchangeable: that is, the potential outcomes are not independent of the group assignment and the unadjusted group difference in outcomes are potentially biased due to selection bias and associated heterogeneous treatment effects (28, 29). In this case, the uplift modeling framework needs to be extended to accommodate confounding, which we illustrate for our case of hemodynamic management presented in Figure 2. As our exposure (hemodynamic management) comprises two joint (numerical) treatment choices—the total intraoperative fluid balance and norepinephrine administration—we first dichotomized the treatment into a control and a treatment group to mimic the two-arm design of a randomized controlled trial (Figure 2A; see Materials and methods below). Next, the causal effect of the dichotomized hemodynamic treatment (T) on PO-AKI (Y) is illustrated in a causal graph and is identified by means of the so-called back-door criterion (Figure 2B) (30).

The back-door criterion allows to derive the variable set required to obtain an unbiased estimate of the causal treatment effect. Based upon this variable set, inverse probability of treatment weighting (IPTW) with associated weights allows to derive a pseudo population in which the set of confounding variables are balanced (Figure 2C). The size of the pseudo population is twice the size of the original cohort and random samples from the pseudo population can be drawn. Based on the sampled pseudo population, outcome models for the control and treatment group are computed to build an uplift model (Figure 2D).

## 3 Materials and methods

### 3.1 Cohort and ethics

Data from a prospectively maintained database of cystectomy procedures at a single tertiary center was used. Patients and procedures from 1 January 2000 to 30 June 2020 were extracted from the database and completed from the patients’ paper charts and anesthetic protocols. Ethical approval was provided by the Ethical Committee of Canton Bern, Switzerland (KEKBE 2016-00660, Chairperson Professor C. Seiler) on 2 June 2016. The need for informed consent was waived.

### 3.2 Surgical technique, perioperative management, and outcomes

Over the past two decades, open cystectomy procedures with urinary diversions have been performed following a standardized surgical technique (31, 32). Induction of anesthesia—consisting of a fentanyl 2 μg⋅kg^–1^ bolus, propofol 2 mg⋅kg^–1^ and rocuronium 0.6–0.9 mg⋅kg^–1^ administration—was performed after insertion of an epidural catheter at the low thoracic level. Maintenance of anesthesia was performed with halogenics.

With respect to the fluids, lactated Ringer’s solution combined with 4% gelatin or 6% hydroxyethyl starch 130/0.4 was administered before the year 2007. Afterward, a protocol-driven restrictive fluid administration was aimed, using a pre-emptive continuous administration of norepinephrine (NE) (around 1–2 μg⋅kg^–1^⋅h^–1^) combined with a fluid maintenance rate of approximately 1–3 mL⋅kg^–1^⋅h^–1^ of lactated Ringer’s solution beginning after initiation of the epidural segmental blockade and anesthesia induction. Blood loss was primarily replaced with lactated Ringer’s solution. Additional fluid administration (albumin, lactated Ringer’s) could be administered at the discretion of the anesthesiologist in charge in case of severe hemorrhage (>20% of the estimated blood volume). Packed red blood cells (PRBCs) were transfused if hemoglobin values decreased below 80 g⋅L^–1^. Assessment of urinary output was not feasible during surgery because of external derivation of the ureter. Postoperative intravenous hydration consisted of 1,000 mL of crystalloids and 500 mL of glucose 5% daily until resumption of normal food intake (33). In case of hypotension, an additional bolus of 250–500 mL of lactated Ringer’s solution was administered. Immediately after surgery, the patients were offered oral clear fluids. A peroral liquid diet and active mobilization were started on postoperative day (POD) 1.

The primary endpoint was the incidence of PO-AKI defined according to the Kidney Disease: Improving Global Outcomes (KDIGO) classification based on changes in plasma creatinine levels (34).

### 3.3 Treatment dichotomization, inverse probability of treatment weighting and causal inference

**FIGURE 3:**
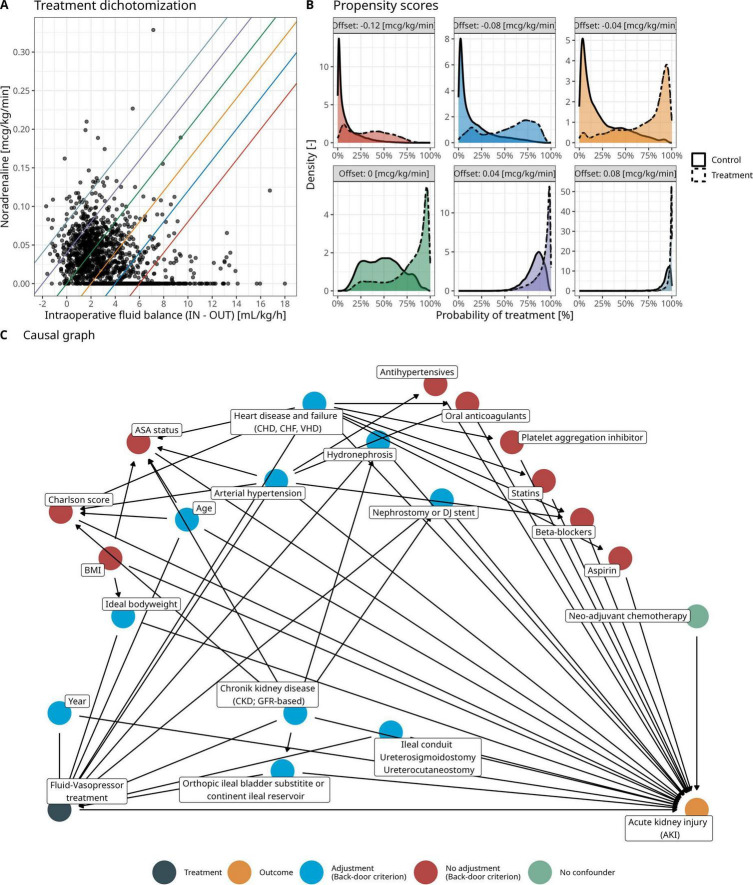
**(A)** Observed and dichotomized hemodynamic treatment in the cohort of this study. Different colors denote different thresholds for treatment dichotomization with a fixed slope (0.02 μg/kg/min per mL/kg/h) and varying offset in norepinephrine administration (ranging from - 0.12 to 0.08 μg/kg/min by increments of 0.04 μg/kg/min). **(B)** Propensity scores for different treatment dichotomization choices to assess overlap between patients in the control and treatment group according to the hemodynamic treatment dichotomization. **(C)** Clinically derived causal graph relating the dichotomized fluid-norepinephrine to postoperative acute kidney injury (refer to Figure 2B). Colors denote the set of adjustment variables identified by the backdoor criterion.

To account for the observed inverse relationship between total intraoperative fluid balance (TIFB, defined as the amount of fluid administered (IN) minus blood loss (OUT)) and NE administration, the joint TIFB-NE regime was dichotomized according to a line with a fixed slope (0.02 μg⋅kg^–1^ min^–1^ per mL⋅kg^–1^⋅h^–1^) and varying offsets in NE administration (ranging from −0.12 to 0.08 μg⋅kg^–1^⋅min^–1^ by increments of 0.04 μg⋅kg^–1^⋅min^–1^) as shown in Figure 3A. The validity of the dichotomization approach was examined by means of the overlap between the propensity scores (Figure 3B) for different dichotomization choices. A logistic regression was used as method for propensity score estimation. Based on the derived propensity scores, we consider the offset choice of −0.04 μg⋅kg^–1^⋅min^–1^ in NE administration as the primary dichotomization choice—other dichotomization choices are only considered for sensitivity analyses. The results of these sensitivity analyses are provided in Supplementary material.

The set of conditioning variables for the IPTW were based on a clinically derived causal directed acyclic graph and application of the backdoor criterion (Figure 3C). Note that the variable year accounts for a possible causal effect of the treatment changes in 2007 on both the fluid-norepinephrine treatment and the primary outcome. Assuming no unmeasured confounding, the positivity of treatment assignment (Figure 3B), exchangeability by means of the backdoor criterion (Figure 3C) and consistency (that the potential outcome for a given dichotomized hemodynamic treatment corresponds to the observed outcome), the causal of effect of the dichotomized fluid-norepinephrine treatment on PO-AKI can be identified.

### 3.4 Uplift and prediction modeling

Based on the IPTW-derived weights, a pseudo-population can be calculated in which the confounding variables are balanced: we achieve so by multiplying the IPTW-weights by a factor of 10,000 and add each patient multiple times according to the 10,000-multiplied weight to a super population. This step is required as the weights are not integers but real numbers. From this super population, a sample can be randomly drawn for the treatment and control group separately—each the size of the original cohort such that the sampled confounder-balanced pseudo-population has the size twice of the original cohort.

An uplift model based on the two-model approach is computed using the sampled pseudo population. Note that for the uplift models, all available predictors can be used (Supplementary Table SM1)—not only the predictors relevant for causal inference. With respect to model formulation, we chose logistic regression and a random forest as underlying statistical models. As benchmark, we further built traditional prediction models for PO-AKI and examined their utility in sorting patients according to expected treatment benefits. The prediction models were based on a logistic regression model and a random forest and included all available variables (Table 1). Given the observational study data, we emphasize that the prediction models are based on the random samples from the IPTW-derived pseudo-population to allow a comparison between the two modeling frameworks.

**TABLE 1:** Cohort characteristics stratified according to postoperative acute kidney injury (PO-AKI; primary outcome).

	PO-AKI	No PO-AKI
	*N* = 321 (21.7%)	*N* = 1,161 (78.3%)
**Demographics and comorbidities**		
Age (years)	69.1 [63.5;76.0]	68.4 [60.3;75.6]
Body Mass Index (kg/m^2^)	27.1 [24.3;30.4]	25.2 [22.6;28.4]
**ASA physical status**		
1	2 (0.6%)	29 (2.5%)
2	131 (40.8%)	601 (51.8%)
3	176 (54.8%)	506 (43.6%)
4	12 (3.8%)	25 (2.1%)
Charlson Comorbidity Index	4 [2;6]	4 [0;5]
**Chronic kidney disease**		
Stage 1	80 (24.9%)	341 (29.4%)
Stage 2	136 (42.4%)	472 (40.7%)
Stage 3	91 (28.3%)	294 (25.3%)
Stage 4	11 (3.4%)	42 (3.6%)
Stage 5	3 (0.93%)	12 (1.03%)
COPD (Yes)	67 (20.9%)	231 (19.9%)
Heart disease and failure (Yes):	125 (38.9%)	338 (29.1%)
Arterial hypertension (Yes)	206 (64.2%)	550 (47.4%)
Hydronephrosis (Yes)	70 (21.8%)	247 (21.3%)
Neoadjuvant chemotherapy (Yes)	58 (18.1%)	172 (14.8%)
**Drugs**		
Oral anticoagulants (Yes)	17 (5.30%)	43 (3.70%)
Statins (Yes)	100 (31.2%)	246 (21.2%)
Antihypertensives (Yes)	192 (59.8%)	467 (40.2%)
Beta-blockers (Yes)	89 (27.7%)	218 (18.8%)
**Platelet aggregation inhibitor:**		
None	258 (80.4%)	1016 (87.5%)
Mono or dual	63 (19.6%)	145 (12.5%)
**Procedure**		
Year of procedure	2012 [2007;2016]	2010 [2006;2015]
Orthopic ileal bladder substitute or continent ileal reservoir (Yes)	157 (48.9%)	593 (51.1%)
Ileal conduit, ureterosigmoidostomy, ureterocutaneostomy (Yes)	166 (51.7%)	575 (49.5%)
Preoperative nephrostomy or DJ stent (Yes)	47 (14.6%)	163 (14.0%)
**Hemodynamic management**		
Intraoperative fluid balance (IN minus OUT; mL/kg/h)	2.4 [1.2;4.1]	3.1 [1.7;5.2]
Norepinephrine (μg/kg/min)	0.03 [0.00;0.05]	0.02 [0.00;0.05]

Further details regarding the cohort are provided in the primary publications.

The models’ clinical utility for patient selection are examined by means of uplift by decile plot and the area under the Qini curve. The model evaluation is based on a random cross-validation in which 60% of the IPTW-pseudo population are used for model building and the remaining 40% for model evaluation. Overall, 500 bootstrap samples of the original data were taken. A super population for each bootstrap sample was derived, from which five random samples were taken. For each of those samples, two random cross-validation samples were drawn to build and evaluate the uplift and prediction models. This large, nested bootstrap framework results in a robust, computationally intensive model evaluation and model metrics are illustrated with mean and 95%-CI. The modeling framework is illustrated in detail in Supplementary Figure SM1.

### 3.5 Summary statistics, missing values, and statistical software

Categorical variables were summarized with counts and frequencies. Numerical variables were summarized with mean and standard deviation in case of normally distributed variables and with median and interquartile range (IQR) otherwise. The analysis was based on a complete-case analysis (*N* = 1,482 from *N* = 1,489).

All computations were performed with R versions 4.2.1 (35). The selection of confounding variables by means of the back-door criterion was performed with the *ggdag* and *dagitty* packages (36, 37). The area under the Qini curve was computed with the *tools4uplift* package (38). The random forest was calculated with the *randomForest* package (39).

## 4 Results

### 4.1 Cohort description

Baseline characteristics and details regarding drug administration, procedure and hemodynamic management are shown in Table 1. Median age was 69 years (IQR: 61–76 years). Most patients had ASA physical status 2 and 3 and the median Charlson Comorbidity Index was 4. Stage 2 chronic kidney disease (CKD) was present in 41%. Median TIFB was 2.9 mL⋅kg^–1^⋅h^–1^ (IQR: 1.6–4.9 mL⋅kg^–1^⋅h^–1^). Median NE administration was 0.02 μg⋅kg^–1^⋅min^–1^ (IQR: 0–0.05 μg⋅kg^–1^⋅min^–1^). Incidence of PO-AKI was 21.7% (95%-CI: 19.6–23.8%).

### 4.2 Average treatment effect

For our primary dichotomization choice, 835 patients were allocated in the control group and 647 patients in the treatment group (Table 2). The incidence of PO-AKI was 25.4% (control group) and 16.8% (treatment group), respectively. Based on IPTW, we derive an ATE of 12.4% (95%-CI: 5.2–19.4%) in favor of a high TIFB / low NE treatment. However, the ATE is sensitive to the choice of hemodynamic treatment dichotomization and the mean estimate can vary from 2.5 to 12.4% (Supplementary material SM2).

**TABLE 2:** Estimates of the average treatment effect and performance metrics of traditional prediction models and uplift models.

(A) Cohort and treatme*n*t		
	Control group	Treatment group
Number of patients	*N* = 835	*N* = 647
Postoperative Acute Kidney Injury (PO-AKI)	212 (25.4%)	109 (16.8%)
Total intraoperative fluid balance (mL/kg/h)	1.74 [1.04;2.55]	5.19 [3.93;6.69]
Norepinephrine (μg/kg/min)	0.04 [0.03;0.06]	0.00 [0.00;0.01]
**(B) Causal inference**		
Average treatment effect (treatment—control)	12.4% (95%-CI: 5.2–19.4%)	
**(C) Discrimination performance (AUROC)**		
Prediction models		
Logistic regression	0.70 (95%-CI: 0.63–0.77)	
Random forest	0.98 (95%-CI: 0.97–0.99)	
Uplift models	Control group	Treatment group
Logistic regression	0.62 (95%-CI: 0.52–0.71)	0.63 (95%-CI: 0.55–0.71)
Random forest	0.85 (95%-CI: 0.79–0.89)	0.82 (95%-CI: 0.77–0.86)
**(D) Uplift modeling (AUQC)**		
**Benchmark**		
Random patient allocation	0.06 (95%-CI: 0.02–0.06)	
Optimal patient allocation	0.48 (95%-CI: 0.43–0.48)	
**Prediction models**		
Logistic regression	0.06 (95%-CI: 0.03–0.06)	
Random forest	0.06 (95%-CI: 0.03–0.06)	
**Uplift models**		
Logistic regression	0.13 (95%-CI: 0.08–0.13)	
Random forest	0.30 (95%-CI: 0.26–0.30)	

(A) Description of the cohort, hemodynamic treatment and incidence of postoperative acute kidney injury (PO-AKI) when hemodynamic treatment is dichotomized (see Materials and methods: shown here is the case for an offset with respect to norepinephrine of −0.4 μg/kg/min.). (B) Discrimination performance of both standard prediction models and uplift models with respect to the primary outcome PO-AKI. Note that the uplift models feature a prediction model separately for the control and the treatment group (Figure 1). (C) Estimate of the average treatment effect of the dichotomized hemodynamic treatment on PO-AKI based on inverse probability of treatment weighting (IPTW; see Materials and methods). (D) Performance of different model types (e.g., prediction models and uplift models) in sorting patients according to treatment benefit as measured by the area under the Qini curve (AUQC; see Materials and methods). The AUQC is also shown for two benchmark scenarios where patients are sorted randomly and in a theoretical, optimal fashion (see Materials and methods). AUROC, Area under the receiver operating characteristic curve; AUQC, Area under the Qini curve (see Materials and methods).

### 4.3 Identification of patients with beneficial treatment effects

The discrimination performances of the prediction models involved in the identification of patients with a beneficial hemodynamic treatment effect (a high TIFB/low NE regime) on PO-AKI are shown in Table 2C. The random forest prediction model demonstrates a significantly higher AUROC than the logistic regression model, both in the traditional prediction case (AUROC of 0.98, 95%-CI: 0.97–0.99, versus 0.70, 95%-CI: 0.63–0.77) and the uplift modeling case. For example, the outcome prediction models for the control group in the two-model uplift model feature an AUROC of 0.62 (95%-CI: 0.52–0.71) and 0.85 (95%-CI: 0.79–0.89) for the logistic regression and the random forest model, respectively. Supplementary Table SM3 illustrates the average distribution of the four response types predicted by the uplift models. Around 80% of the patients are predicted to be of the *Sure Thing* type. Around 10–15% are predicted to be of the *Persuadable* type, that is, benefitting from a high TIFB and low NE treatment. There are only few patients (∼1%) predicted to suffer from PO-AKI independent of treatment choice (*Lost Causes*).

**FIGURE 4:**
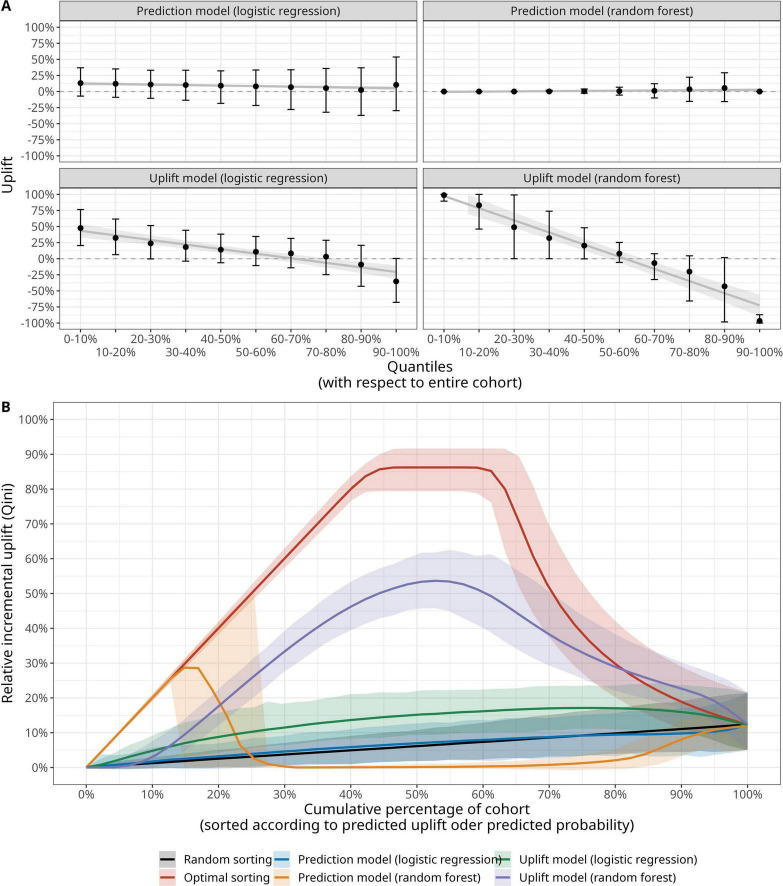
Evaluation of outcome prediction models and uplift models with **(A)** decile plot (mean, 95% confidence interval and a locally estimated scatterplot smoothing (LOESS) estimate of the relationship of uplift versus decile) and **(B)** area under the Qini curve (AUQC), where the solid line denotes the mean value and the shading corresponding the 95% confidence interval derived from a large bootstrap sample (see Materials and methods and Supplementary Figure SM1).

The uplift by decile plots for the different models are shown in Figure 4A. In contrast to the traditional prediction models, the uplift models demonstrate ability in sorting the patients according the expected treatment benefit as they demonstrate a monotonically decreasing uplift from the decile with highest predicted uplift (0–10%, left part of each uplift by decile panel) to the decile with lowest predicted uplift (90–100%, right part of each uplift by decile panel). The uplift models feature higher AUQCs than the traditional prediction models (Table 2D): the random forest uplift model features an AUQC of 0.30 (95%-CI: 0.26–0.30), thus providing a clinical benefit when compared to the random sorting strategy with an AUQC of 0.06 (95%-CI: 0.02–0.06). The AUQC of the traditional prediction models is close the AUQC of the random sorting strategy.

The corresponding Qini curves are illustrated in Figure 4B and reveal an interesting pattern with respect of the relative performance of traditional prediction models and uplift models. A prediction model for PO-AKI based on a random forest is able to identify the top 15% patients of the underlying population with the highest expected treatment benefit. In this case, the model predicts the highest likelihood for a PO-AKI-free recovery for patients in the treatment group and the Qini curve is identical to the optimal Qini curve for these patients. However, the random forest prediction model fails to sort the remaining patients according to expected treatment benefit and the uplift models provide larger clinical benefit. While the clinical benefit of the uplift models—in particular when based on a random forest—is robust with respect to the choice of fluid-norepinephrine dichotomization, the ability of traditional prediction models in identifying patients with the largest benefit strongly depends on the way patients are allocated to the control and treatment group (Supplementary Figures SM3–SM7).

## 5 Discussion

In the search for new, personalized approaches to tailor clinical decision-making and associated treatments closer to each patient, the domain of individualized treatment effect modeling (or uplift modeling) has gained increasing interest over the past years, both in terms of theoretical developments and practical implementations (11, 16, 40–44). While traditional prediction models (or so-called response models) predict the probability (or risk) of the outcome Y for each patient, uplift models compute the incremental change in outcome of an alternative treatment with respect to some control treatment (ΔY/ΔT) for each unit (25). Uplift modeling thus provides a clinically relevant addition to traditional risk stratification by involving the potential impact of actual treatment decisions on the outcome of an individual patient—thus moving from predictive to so-called prescriptive analytics (13). This prescriptive clinical reasoning involves the consideration of causal inference notions, in particular the potential outcome framework and heterogeneous treatment effects (11).

In this context, the Predictive Approaches to Treatment effect Heterogeneity (PATH) Statement was recently published to promote the conduct of, and provide guidance for the predictive analyses of heterogeneity of treatment effects (HTE) in clinical trials (45). In particular, the PATH statement introduces the notion of predictive HTE. A recent article highlighted the common objectives shared by HTE and uplift modeling (19). A systematic benchmarking study recently suggested to use multiple methods for estimating individualized treatment effects from both research streams (46).

Here, we briefly introduced the key concepts of uplift modeling with an emphasis on the evaluation of the uplift models by means of the Qini curve and its associated AUQC. The AUQC provides a valuable metric familiar to the AUROC in standard prediction modeling for binary outcomes. Importantly, the AUQC provides an intuitive benchmark scenario where patients are randomly allocated to either the control or the alternative treatment of interest. We note that traditional clinical prediction modeling plays in integral part in the so-called two-way modeling approach to uplift modeling (Figures 1, 2). In this approach, traditional prediction models constitute the knots and bolts of an uplift model. Table 1 demonstrates that the underlying prediction models feature an adequate discriminatory performance for the application considered here, in particular the models based on a random forest.

In this study, we presented the first application of uplift modeling for a cohort of patients scheduled for cystectomies and urinary diversions with PO-AKI as outcome. The model building process involved several technical steps as the available data derived from an observational study and not from a randomized controlled trial (the usual standard for the computation of individualized treatment effects) (21). In addition, we considered a joint treatment of two continuous variables (TIFB and NE) and performed a clinically comprehensible—yet still subjective—treatment dichotomization.

A key result is that we found evidence of the uplift model’s ability to sort patients according to the expected benefit from either a high TIFB and low NE regime or vice versa. This finding derives from the models AUQC values, which were significantly higher than those derived from a random sorting strategy (Figure 4). Our analysis further allowed us to compare traditional prediction modeling approaches with the uplift framework. This comparison is of clinical importance as one could also simply sort the patients according to their baseline PO-AKI risk—irrespective of consideration of possible hemodynamic treatment choices. In this study, a random forest prediction model identified the 15% of patients with the lowest PO-AKI risk: these patients were treated with a high TIFB/Low NE regime and showed a PO-AKI free recovery, thus following the optimal Qini curve. However, for the remaining patients, the choice of treatment does matter—and for these patients the uplift modeling framework is clinically more useful for patient identification than the prediction model.

Of interest is the finding that the models based on a random forest demonstrated higher prediction ability both in terms of AUROC and AUQC, which could not be expected a priori given the limited number of predictors and moderate sample size, warranting further sensitivity analyses (47). Thus, conditional on the cohort and modeling approaches of this study, the uplift framework provides an overall higher clinical benefit in examining optimal fluid-norepinephrine treatment choices for the entire patient cohort than prediction models for baseline PO-AKI risk.

Despite the depth and variety of statistical methods involved in building an uplift model from an observational dataset, the models derived here have a strong applicability and potential in clinical practice. For example, the uplift models’ capacity to guide fluid-norepinephrine treatment allocation with respect to PO-AKI could be validated in a prospective study. In addition, the output of an uplift model can be interpreted in a straightforward fashion with respect to the four response types. Positive uplift values close to 1 indicate that a patient is likely to be of the *Persuadable* type, whereas an uplift values close to −1 designates a *Do-not-disturb* type. However, there is ambiguity for uplift values close to zero as these values can relate to both a *Lost-cause* and a *Sure Thing* type of patient. We emphasize here again these response types are unobservable and that group-level performance metrics such as the AUQC are required to evaluate an uplift model.

### 5.1 Limitations

This study features inherent limitations. First, only data from a single center was used and future multi-center studies are required to examine the external validity and robustness of our findings. Second, the uplift framework as implemented here features only a simple, two-model approach. In the future, more complex frameworks including Bayesian, Machine and Deep Learning approaches would be possible (41, 48). Third, the causal inference analysis (e.g., the causal graph) was based on available data and not a pre-defined collection of variables specifically chosen for causal analysis. Ideally, the causal graph with the application-relevant variables should be clinically derived prior to the study. Thus, future studies with broader population characteristics and different types of intervention would define the clinical variables relevant for causal inference by involving the subject-matter knowledge of different clinical specialties. Fourth, the clinical utility of the uplift models was examined by means of uplift by decile plots and Qini curves. Future studies could employ more advanced metrics, e.g., with reduced variance (49). Fifth, the TIFB is the sum of sequential decision making which likely results in time-dependent treatment-confounder feedback. In these circumstances, dedicated methods beyond the scope of the study are required (28). Sixth, the uplift framework as featured in this study considers only treatment benefits and does not incorporate possible treatment-related harms, which could potentially alter the sorting of patients. Finally, the uplift framework does not systematically incorporate risk preferences expressed as probabilistic treatment thresholds for clinical decision making such as featured in a decision curve analysis (50).

## 6 Conclusion

Uplift modeling provides a clinically relevant step toward personalized medicine by considering the incremental treatment benefit of a specific patient, thus moving from a predictive toward a prescriptive risk assessment. In this study, we applied the uplift framework to an observational cohort of patients scheduled for cystectomy and urinary diversion procedures and demonstrated its clinical utility in sorting patients according to the expected treatment benefit from either a high total intraoperative fluid balance / low norepinephrine regime or a low total intraoperative fluid balance / high norepinephrine regime. The uplift modeling approach provided higher clinical utility than a traditional prediction modeling framework in examining the optimal hemodynamic treatment (combining fluid and vasopressor) allocation with respect to a PO-AKI free recovery in the overall cohort. However, the ability of the uplift models to optimize hemodynamic treatment allocation choices needs to be evaluated in future, prospective studies.

## Data Availability

The datasets presented in this article are not readily available because the datasets generated and/or analyzed during the current study are not publicly available given the current status of the ethical approval but may be available from the corresponding author on reasonable request, including a formal ethics approval from the corresponding institution. Requests to access the datasets should be directed to Patrick.Wuethrich@insel.ch.
