# Supplementary material for: Uplift modeling to determine which fluid-norepinephrine regime results in a postoperative acute kidney injury-free recovery in patients scheduled for cystectomy and urinary diversions
Source: Front Med (Lausanne). 2025 Jun 11;12:1542797. doi: 10.3389/fmed.2025.1542797 (PMC12187774; doi:10.3389/fmed.2025.1542797)
Supplement: Supplementary file 1 [file Data_Sheet_1.docx]

Supplementary Material

Uplift modeling to determine which fluid-norepinephrine regime results in a postoperative acute kidney injury-free recovery in patients scheduled for cystectomy and urinary diversions

**Markus Huber^1*^, Marc A. Furrer^1,2,3^, Francois Jardot^1^, Patrick Y. Wuethrich^1^**

^1^ Department of Anaesthesiology and Pain Medicine, Inselspital, Bern University Hospital, University of Bern, Bern, Switzerland

^2^ Department of Urology, Inselspital, Bern University Hospital, University of Bern, Bern, Switzerland

^3^ Department of Urology, Solothurner Spitäler AG, Olten, Switzerland

*** Correspondence:**Markus Huber, Dr. sc. ETH. Department of Anaesthesiology and Pain Medicine, University Hospital Bern, Inselspital, CH-3010 Berne, Switzerland. Phone +41 31 664 12 15, [markus.huber@insel.ch](mailto:markus.huber@insel.ch)

**
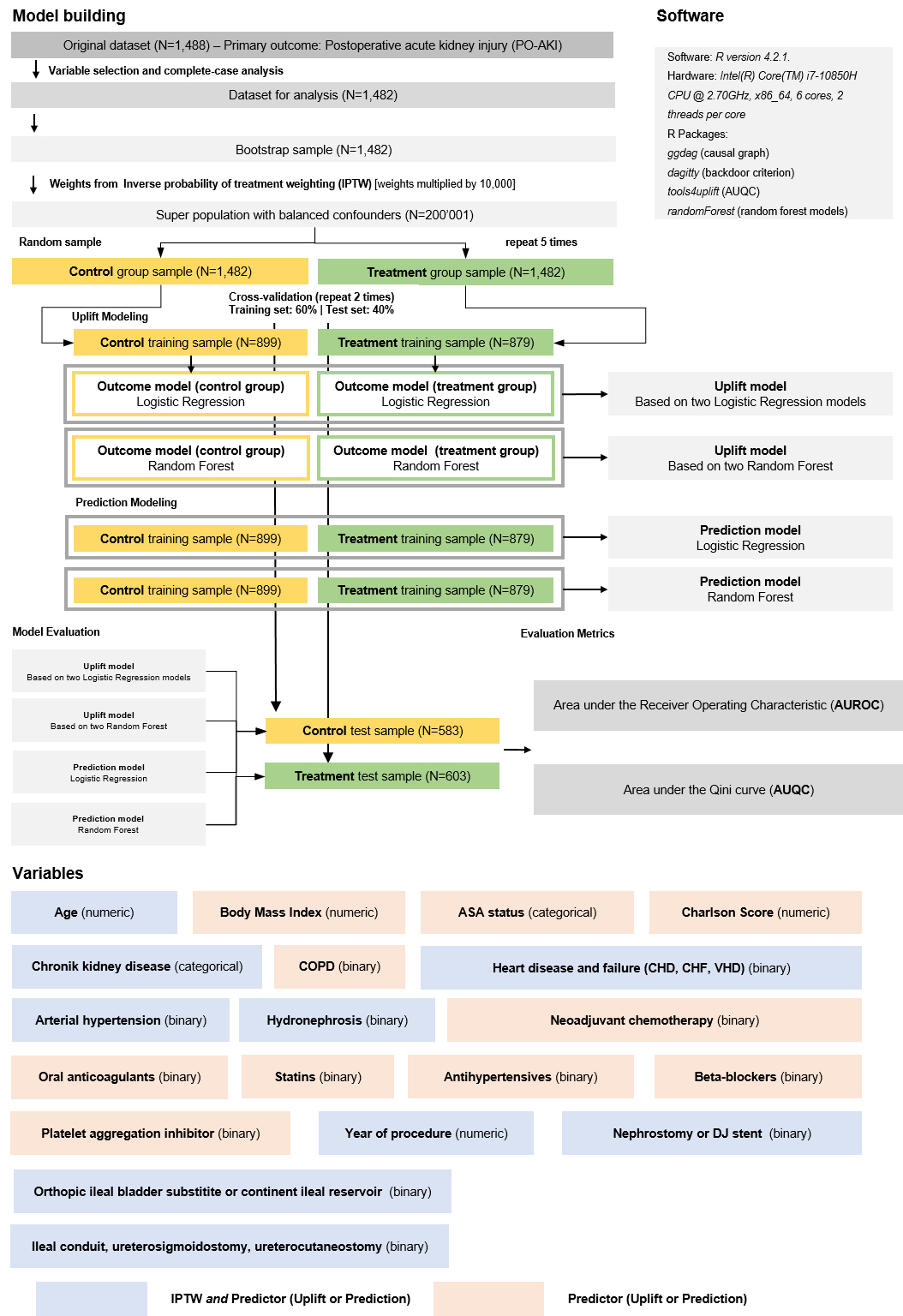
**

**Supplementary Figure SM1.** Illustration of the model building and evaluation process of this study.

| 1. *Norepinephrine offset: -0.12 µg/kg/min* |  |  |
| --- | --- | --- |
|  | **Control Group** | **Treatment Group** |
| **Number of patients:** | N=1282 | N=200 |
| **Postoperative Acute Kidney Injury** (AKI): | 301 (23.5%) | 20 (10.0%) |
| **Total fluid balance** (mL/kg/h) | 2.50 [1.45;4.02] | 7.67 [6.62;9.40] |
| **Norepinephrine** (*µg/kg/min*) | 0.03 [0.01;0.05] | 0.00 [0.00;0.00] |
| **Average treatment effect** (Treatment – Control) | 12.4% (95%-CI: 2.0% - 19.6%) | |
| 1. *Norepinephrine offset: -0.08 µg/kg/min* |  |  |
|  | **Control Group** | **Treatment Group** |
| **Number of patients:** | N=1081 | N=401 |
| **Postoperative Acute Kidney Injury** (AKI): | 268 (24.8%) | 53 (13.2%) |
| **Total fluid balance** (mL/kg/h) | 2.16 [1.33;3.21] | 6.28 [5.23;7.87] |
| **Norepinephrine** (*µg/kg/min*) | 0.04 [0.02;0.06] | 0.00 [0.00;0.00] |
| **Average treatment effect** (Treatment – Control) | 9.7% (95%-CI: 0.7% - 17.0%) | |
| 1. *Norepinephrine offset: -0.04 µg/kg/min* |  |  |
|  | **Control Group** | **Treatment Group** |
| **Number of patients:** | N=835 | N=647 |
| **Postoperative Acute Kidney Injury** (AKI): | 212 (25.4%) | 109 (16.8%) |
| **Total fluid balance** (mL/kg/h) | 1.74 [1.04;2.55] | 5.19 [3.93;6.69] |
| **Norepinephrine** (*µg/kg/min*) | 0.04 [0.03;0.06] | 0.00 [0.00;0.01] |
| **Average treatment effect** (Treatment – Control) | 12.4% (95%-CI: 5.2% - 19.4%) | |
| 1. *Norepinephrine offset: 0 µg/kg/min* |  |  |
|  | **Control Group** | **Treatment Group** |
| **Number of patients:** | N=472 | N=1010 |
| **Postoperative Acute Kidney Injury** (AKI): | 120 (25.4%) | 201 (19.9%) |
| **Total fluid balance** (mL/kg/h) | 1.39 [0.67;2.01] | 4.07 [2.67;5.91] |
| **Norepinephrine** (*µg/kg/min*) | 0.05 [0.04;0.07] | 0.01 [0.00;0.03] |
| **Average treatment effect** (Treatment – Control) | 2.5% (95%-CI: -2.8% - 8.4%) | |
| 1. *Norepinephrine offset: 0.04 µg/kg/min* |  |  |
|  | **Control Group** | **Treatment Group** |
| **Number of patients:** | N=143 | N=1339 |
| **Postoperative Acute Kidney Injury** (AKI): | 40 (28.0%) | 281 (21.0%) |
| **Total fluid balance** (mL/kg/h) | 0.92 [0.17;1.63] | 3.20 [1.87;5.18] |
| **Norepinephrine** (*µg/kg/min*) | 0.08 [0.06;0.10] | 0.02 [0.00;0.04] |
| **Average treatment effect** (Treatment – Control) | 9.8% (95%-CI: 0.8% - 19.4%) | |
| 1. *Norepinephrine offset: -0.12 µg/kg/min* |  |  |
|  | **Control Group** | **Treatment Group** |
| **Number of patients:** | N=34 | N=1448 |
| **Postoperative Acute Kidney Injury** (AKI): | 9 (26.5%) | 312 (21.5%) |
| **Total fluid balance** (mL/kg/h) | 1.03 [0.05;2.06] | 2.95 [1.65;4.96] |
| **Norepinephrine** (*µg/kg/min*) | 0.11 [0.10;0.14] | 0.02 [0.00;0.05] |
| **Average treatment effect** (Treatment – Control) | 10.4% (95%-CI: -10.6% - 29.4%) | |

**Supplementary Table SM2.** Treatment allocation, hemodynamic treatment and average treatment effect for different dichotomization choices as expressed different offsets in norepinephrine administration.

|  | **Uplift Model** | |
| --- | --- | --- |
|  | **Logistic Regression** | **Random Forest** |
| **Response Type** |  |  |
| Lost cause | 16/1,188 (1.3%) | 9/1,187 (0.8%) |
| Do-not-disturb | 82/1,188 (6.9%) | 75/1,187 (6.3%) |
| Persuadable | 15/1,188 (9.8%) | 171/1,187 (14.4%) |
| Sure thing | 974/1,188 (82.0%) | 932/1,187 (78.5%) |

**Supplementary Table SM3.** Predicted response types in the validation set. The number of response types are averaged across the cross-validation procedure (Supplementary Table SM1). The response types are derived by dichotomization of the probabilistic output of the two prediction models on which the two-way uplift model is based (refer to Fig. 1 of the main manuscript).

**
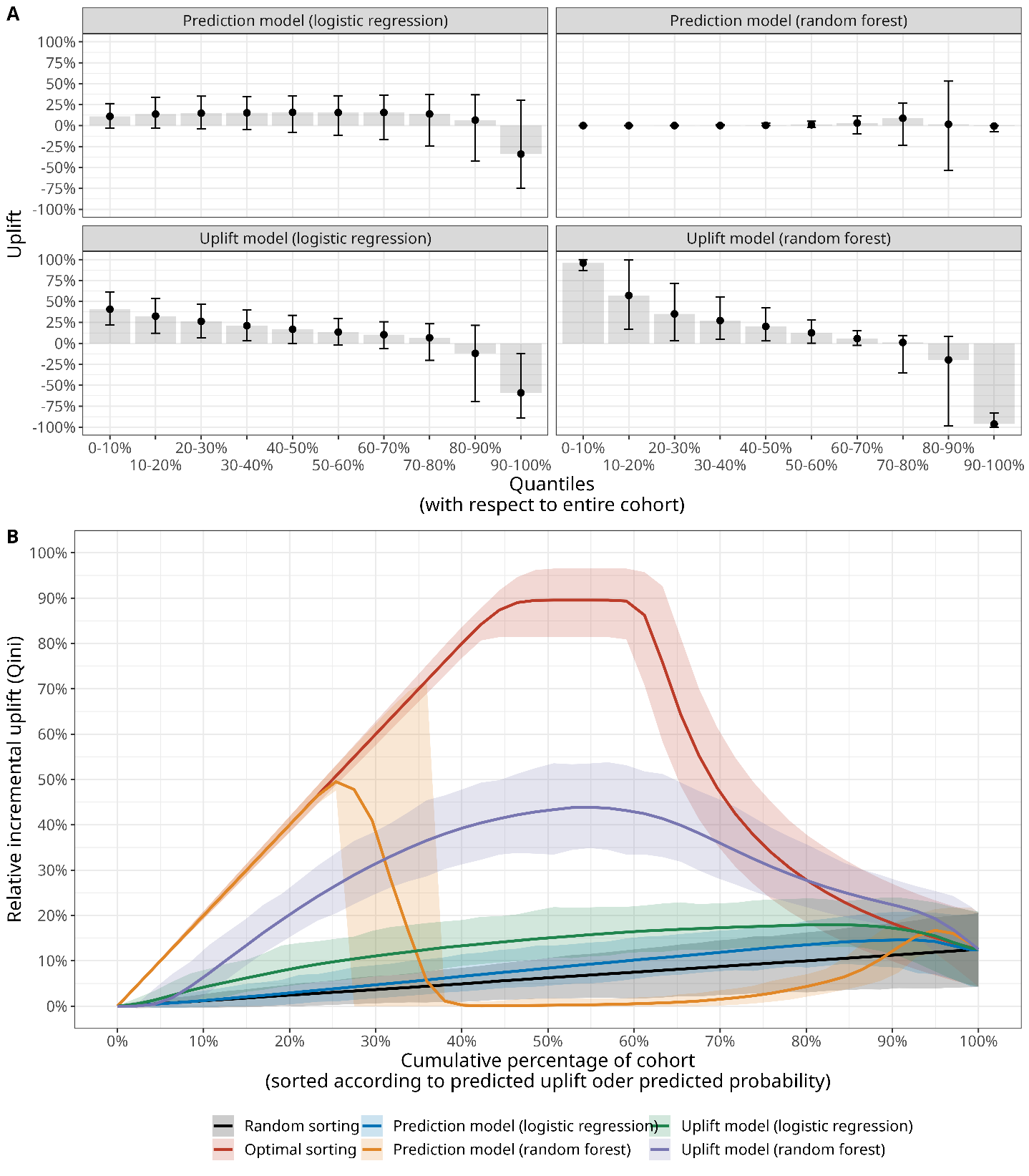
**

**Supplementary Figure SM3.** Similar to Figure 4 of the main manuscript, but for treatment dichotomization with an norepinephrine offset of -0.12 µg/kg/min. Treatment allocation, hemodynamic treatment and average treatment effect for different dichotomization choices as expressed different offsets in norepinephrine administration.


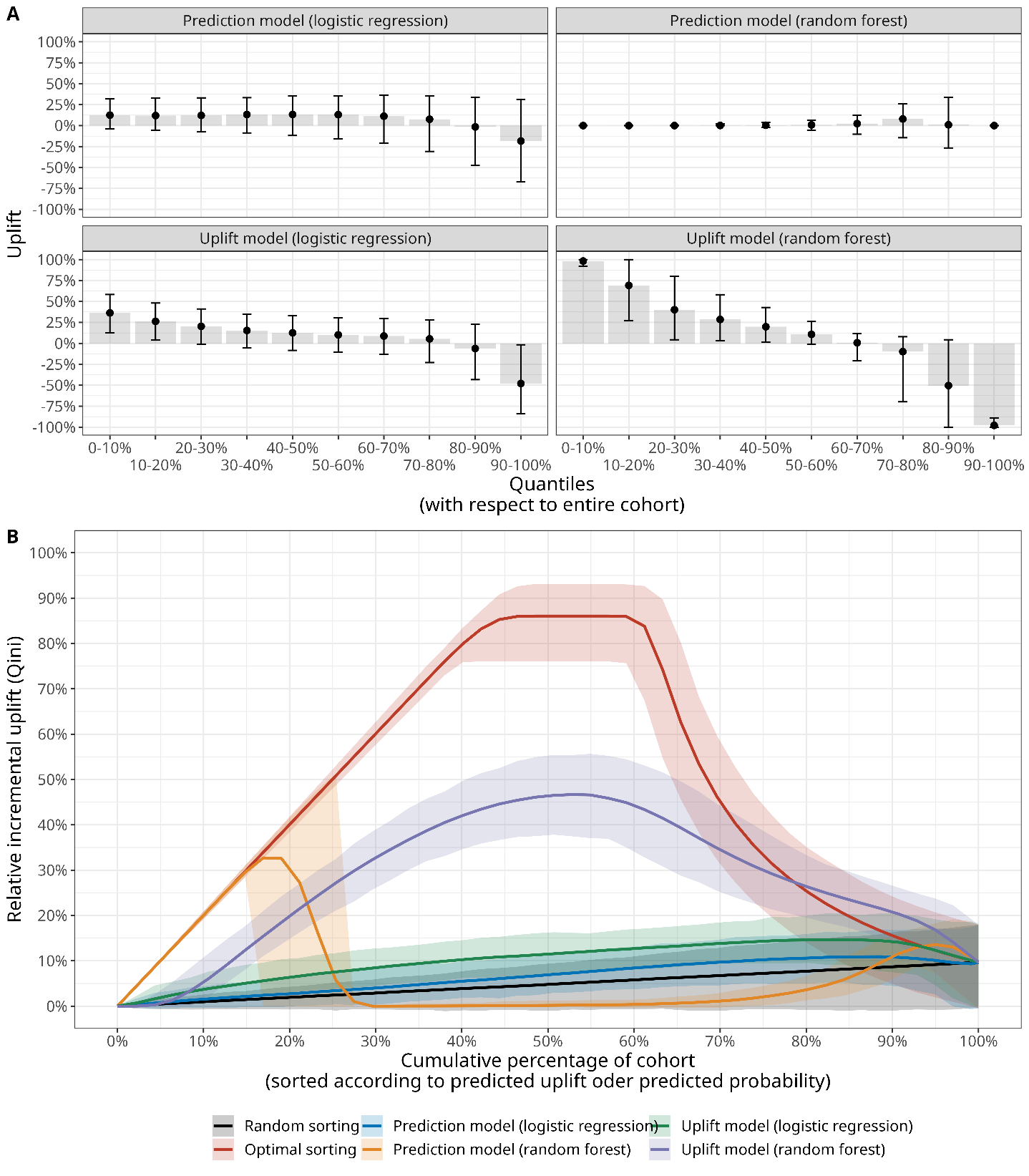


**Supplementary Figure SM4.** Similar to Figure 4 of the main manuscript, but for treatment dichotomization with an norepinephrine offset of -0.08 µg/kg/min. Treatment allocation, hemodynamic treatment and average treatment effect for different dichotomization choices as expressed different offsets in norepinephrine administration.


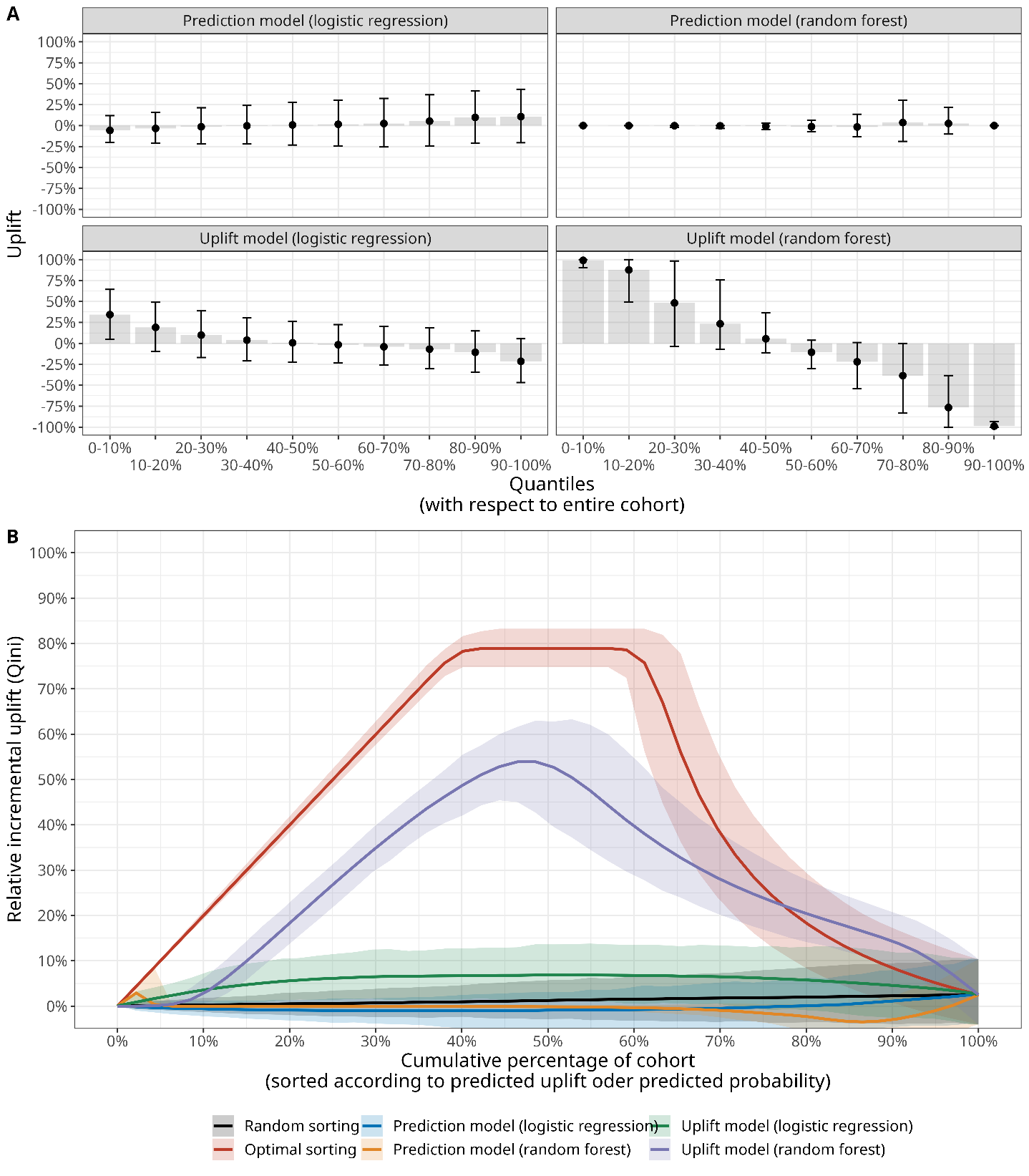


**Supplementary Figure SM5.** Similar to Figure 4 of the main manuscript, but for treatment dichotomization with an norepinephrine offset of 0 µg/kg/min. Treatment allocation, hemodynamic treatment and average treatment effect for different dichotomization choices as expressed different offsets in norepinephrine administration.


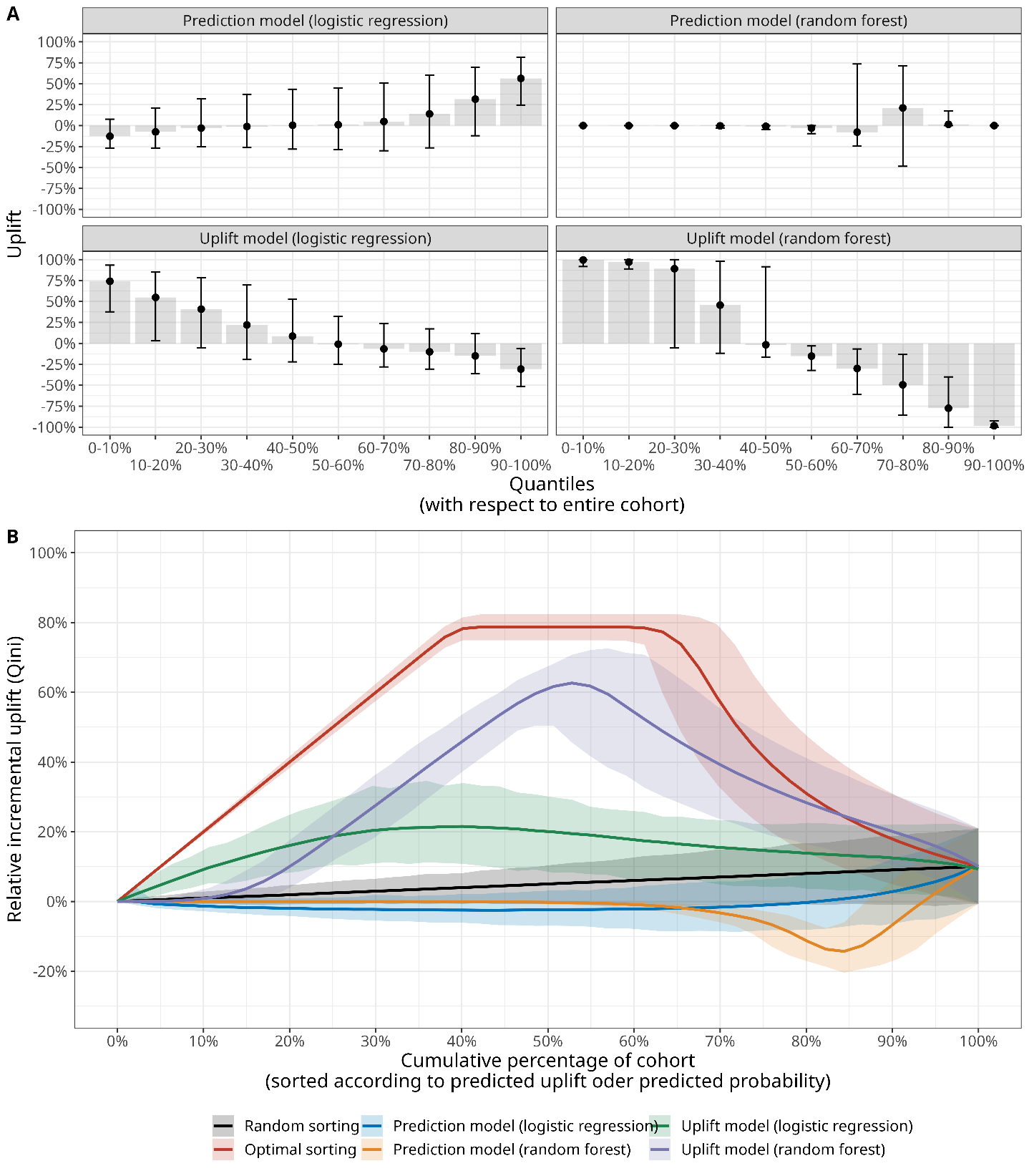


**Supplementary Figure SM6.** Similar to Figure 4 of the main manuscript, but for treatment dichotomization with an norepinephrine offset of 0.04 µg/kg/min. Treatment allocation, hemodynamic treatment and average treatment effect for different dichotomization choices as expressed different offsets in norepinephrine administration.


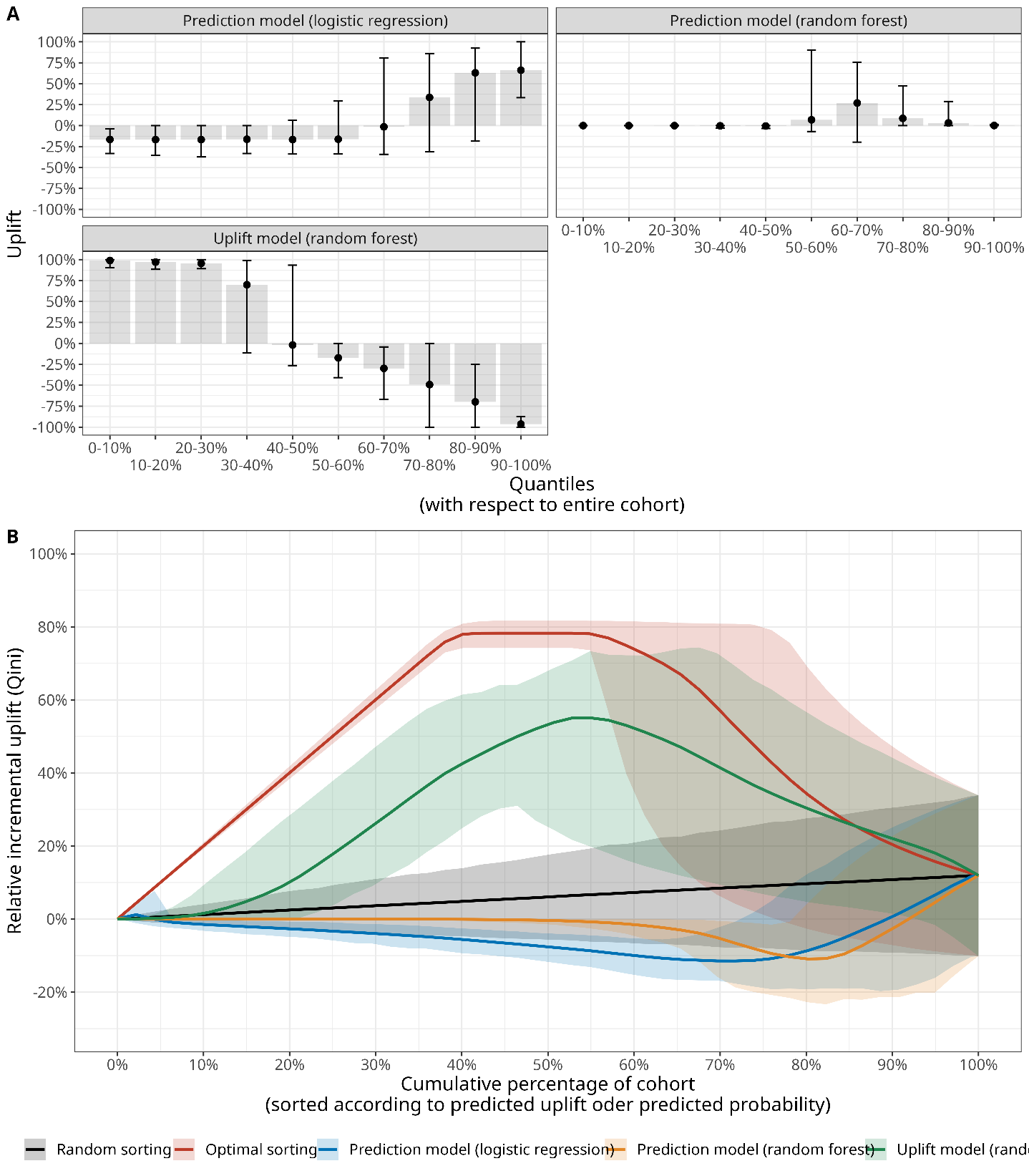


**Supplementary Figure SM6.** Similar to Figure 4 of the main manuscript, but for treatment dichotomization with an norepinephrine offset of 0.08 µg/kg/min. Treatment allocation, hemodynamic treatment and average treatment effect for different dichotomization choices as expressed different offsets in norepinephrine administration. The uplift model based on the logistic regression did not converge – likely due to the small sample size of the control group and the associated risk of perfect separation in some of the categorical variables.
